# Asprosin response in hypoglycemia is not related to hypoglycemia unawareness but rather to insulin resistance in type 1 diabetes

**DOI:** 10.1371/journal.pone.0222771

**Published:** 2019-09-19

**Authors:** Jan Benedikt Groener, Aikaterini Valkanou, Zoltan Kender, Jan Pfeiffenberger, Lars Kihm, Thomas Fleming, Peter Paul Nawroth, Stefan Kopf

**Affiliations:** 1 Department of Medicine I: Endocrinology and Clinical Chemistry, University Hospital Heidelberg, Heidelberg, Germany; 2 Deutsches Zentrum für Diabetesforschung (DZD) e.V., Munich-Neuherberg, Germany; 3 Medicover München Neuroendokrinologie, Munich, Germany; 4 Department of Medicine IV: Gastroenterology and Hepatology, University Hospital Heidelberg, Heidelberg, Germany; 5 Joint-IDC, Institute for Diabetes and Cancer at Helmholtz Zentrum Munich and University of Heidelberg, Munich-Neuherberg and Heidelberg, Germany; West Virginia University, UNITED STATES

## Abstract

Asprosin is a counter-regulatory hormone to insulin which plays a role in fasting. It may therefore also play a role in hypoglycaemia unawareness, which has been subsequently examined in this pilot study. Intravenous glucose tolerance test was used to induce controlled hyperglycemia whereas a hyperinsulinemic clamp test was used to induce a controlled hypoglycaemia in 15 patients with diabetes type 1, with and without hypoglycaemia unawareness. Changes in asprosin plasma levels did not differ between patients with and without hypoglycaemia unawareness. However, nine patients with insulin resistance as well as higher liver stiffness values and low-density lipoprotein but lower high-density lipoprotein levels did not show the expected increase in asprosin plasma levels during hypoglycemia. Therefore, insulin resistance and alterations in liver structure, most likely early stages of non-alcoholic fatty liver disease, seem to be relevant in type 1 diabetes and do not only lead to elevated plasma levels of asprosin, but also to a blunted asprosin response in hypoglycemia.

## Introduction

Asprosin is a recently described protein hormone, produced in the white adipose tissue [1]. It is induced by fasting and is believed to act on the liver, leading to a rapid release of glucose into the circulation leading to compensatory insulin production [1, 2]. The liver-related glucose release into the blood circulation is crucial for brain function as well as overall survival during fasting [3]. In addition, a compensatory rise of asprosin is expected during a hypoglycemic episode [1, 2]. Hypoglycemic episodes are one important cause for the increased mortality in diabetes mellitus type 1, since they can lead to cardiac arrhythmias, stroke, or other potentially lethal complications [4–7]. Therefore, hypoglycaemia unawareness, in which warning symptoms at early phases of hypoglycaemia are reduced or missing, is a dangerous complication which can develop with age, duration of the disease, and lower HbA1c [8].

However, it remains unknown whether hypoglycemia induces a fast rise in asprosin and whether a blunted asprosin release is part of hypoglycaemia unawareness. Therefore, the aim of this pilot study was to investigate the association between low and high glucose levels and asprosin response in patients with type 1 diabetes, with and without hypoglycaemia unawareness.

## Methods

### Study design

Study participants were recruited between May 2017 and August 2019 through the Clinical Study Center for Diabetes Research of the University Hospital Heidelberg. People interested in study participation contacted the Clinical Study Center via phone or e-mail. The study was performed in accordance with the Declaration of Helsinki and was approved by the local ethics committee (Ethikkommission der medizinischen Fakultät der Universität Heidelberg, ethics number S-550/2016, ClinicalTrials.gov Identifier NCT03358121). All participants gave written informed consent. Patients with diabetes mellitus type 1 either with or without known hypoglycaemia unawareness were included. Hypoglycemia unawareness was defined by multiple documented episodes of blood glucose levels below 70 mg/dl without concomitant autonomic warning symptoms [9]. No patients with known liver diseases were included. The full list of inclusion and exclusion criteria is shown in S1 and S2 Tables in the supporting information.

Basic testing for evaluation of late diabetic complications was carried out in one day and included various non-invasive clinical routine tests such as ECG, heart rate variability in inspiration and exspiration as well as lying and upright, and angle-brachial-index (ABI) as screening parameter for peripheral artery disease. Ultrasound of the carotid arteries, kidneys including perfusion, and liver was performed. Additionally, lung function testing, measurement of skin auto-fluorescence as surrogate parameter for accumulation of advanced glycation end products, 24-hour blood pressure measurement, liver stiffness evaluated by transient elastography (FibroScan®, Echosens™ Germany, Wachtberg, Germany), and retinal photography were carried out. Moreover, neuropathy symptom score (NSS) and neuropathy deficit score (NDS) were calculated to screen for diabetic polyneuropathy as described before [10], with ≥3 points in one or both scores clinically indicating diabetic polyneuropathy. On a second day within four weeks after basic testing, routine blood draw in a fasting state, intravenous glucose tolerance test, and hyperinsulinemic clamp examination were performed in sequence. Creatinine values, estimated glomerular filtration rate (eGFR) [11], and urinary albumin/creatinine-ratio were used to screen for diabetic nephropathy.

### Intravenous glucose tolerance test and hypoglycemic hyperinsulinemic clamp study

Intravenous glucose tolerance test (ivGTT) was performed as previously described, whereas a modified version of a hyperinsulinemic clamp study with an additional glucose target of 60–70 mg/dl was performed [12–15]. Participants were invited in the morning after a fasting period of at least 12 hours. Therefore, patients injected the usual dose of basal insulin the night before the study and measured their blood glucose levels in the morning, and corrections by use of the patients’ short acting insulin were made by the patients themselves when necessary. Target glucose levels at baseline of the clamp study were between 100 and 200 mg/dl. Initially, blood was drawn to determine plasma glucose, insulin (both basal insulin and insulin given during the clamp were detected), C-peptide, adrenaline, noradrenaline, glucagon, cortisol, ACTH, and asprosin as well as routine blood tests. Afterwards, ivGTT was performed by intravenous bolus injection of 1 ml/kg of glucose 30% in order to reach peak glucose levels above 250 mg/dl. Serum glucose was measured regularly over a one-hour-period [15]. Additionally, insulin, C-peptide, and asprosin levels were determined ten minutes after injection of the glucose and after one hour at the end of the ivGTT. This test was used to determine the pancreatic insulin reserve and to monitor changes in plasma levels of asprosin during hyperglycemia. Afterwards, a hyperinsulinemic clamp was performed by continuous intravenous infusion of glucose 20% (variable infusion rate according to blood glucose levels and respective targets) and insulin (normal insulin 1.5 mU / kg body weight) in order to set serum glucose levels manually to a target value of 90 mg/dl for a total duration of 1 hour. During this hour, blood was drawn again for evaluation of the above-mentioned hormone levels. Afterwards, the blood glucose target level was lowered to 60–70 mg/dl and was reached by reducing the infusion rate of glucose 20%, in order to draw blood for determination of the above-mentioned hormone levels again. This glucose target was chosen to evaluate changes in plasma levels of asprosin during hypoglycaemia. Lower levels were not chosen due to higher risks for the participants. Catecholamines and cortisol were measured to evaluate stress levels, whereas glucagon was measured due to its similar physiological role to asprosin. Since this clamp was not specifically designed for evaluating insulin sensitivity, whole-body insulin sensitivity was calculated by glucose infusion rate during the last 30 minutes of the clamp study normalized to fat-free mass as described before [16].

### Laboratory analyses

Glucose measurements during ivGTT and clamp testing were performed using point of care testing (Nova Biomedical StatStrip^®^, Nova Biomedical GmbH, Mörfelden-Walldorf, Germany) from venous blood. Routine laboratory tests like HbA1c, liver function tests, albuminuria, as well as hormone analyses of ACTH, cortisol, adrenaline, noradrenaline, glucagon, insulin, and c-peptide were performed in the accredited central laboratory of the University Hospital Heidelberg according to standard protocols. Blood was cooled when necessary and sent to the laboratory immediately after blood-draw. ACTH was measured by chemiluminescence assay, adrenaline and noradrenaline by high-pressure liquid chromatography, cortisol by luminescence immunoassay, glucagon by radio-immunoassay, insulin by electro-chemiluminescence assay, and c-peptide by enzyme immunoassay.

For asprosin measurements, EDTA plasma was used and immediately frozen at minus 80°C after centrifugation at four °C and 530 g for ten minutes. Asprosin values were analyzed using a commercially available ELISA kit (Human Asprosin (ASPRO) ELISA Kit, Wuhan Abebio Science Co., Ltd, Wuhan, China, Code: AE26043HU) in the experimental laboratory of the Department Medicine I at the University Hospital Heidelberg [17]. Samples were handled strictly according to the manufacturer's protocol.

### Statistical analyses

The a-priory calculation of a sample size was not possible for this pilot study since differences in asprosin levels in patients with and without hypoglycemia unawareness had not been studied before.

Non-parametric statistical testing as well as descriptive analyses were used. For independent group comparisons, Mann-Whitney-U test was performed, while correlations were calculated using Spearman Correlation Coefficient. For descriptive analyses, median and range was given.

Statistical testing was performed using IBM SPSS Version 23.0 (IBM Corporation Deutschland, Ehningen), while figures were created using GraphPad Prism Version 7 (GraphPad Software, La Jolla, CA; USA).

## Results

15 patients with diabetes mellitus type 1 were included in this clinical mono-centric pilot study. Median age was 52 (range 29–75) years. Eight women and seven men were included. Median duration of diabetes mellitus was 24 (2–51) years. Four patients had diabetic retinopathy, four patients showed signs of symptomatic polyneuropathy, one patient showed signs of diabetic nephropathy, and one showed cardiac autonomous neuropathy. No patients showed signs for peripheral artery disease. Seven of the 15 patients included had known hypoglycemia unawareness. Median diabetes duration did not differ between patients with and without hypoglycemia unawareness (26 vs. 22 years). 13 of the 15 patients showed increases in adrenalin or noradrenalin during hypoglycemia, irrespective of hypoglycemia unawareness, while there were no associations between the degrees of asprosin increase with alterations in the other hormones examined. Full patient characteristics are given in Table 1 and complete raw data can be found in S3 Table in the supporting information.

**Table 1:** Patients' characteristics and group comparison between patients with and without asprosin increase during hypoglycemic phase.

	Group with asprosin increase (n = 6)	Group without asprosin increase (n = 9)	
Gender (m/f)	3/3	4/5	ns
Age (years)	50.5 (30–66)	56 (29–75)	ns
BMI (kg/m^2^)	24.4 (20.1–31.3)	25.4 (21–38.9)	ns
Smoking	0	2	ns
Diabetes duration (years)	22 (12–36)	30 (2–51)	ns
HbA1c (%) HbA1c (mmol / mol)	7.0 (5.9–9.0) 53.0 (41.0–74.9)	7.6 (6.5–8.5) 59.6 (47.5–69.4)	ns ns
uACR (mg / g creatinine)	2.1 (0.4–8.4)	3.0 (0.7–61.3)	ns
NSS	0 (0–6)	0 (0–5)	ns
NDS	0 (0–10)	2 (0–6)	ns
Glucose infusion rate (ml / min)	100 (65–140)	60 (10–150)	p = 0.066
hsCRP (mg / l)	1.75 (0.15–12.4)	1.86 (0.16–7.93)	ns
Creatinine (mg / dl)	0.76 (0.54–1.06)	0.78 (0.62–1.56)	ns
eGFR CKD-EPI (ml/min*1•73m^2^)	102 (60–127)	101 (43–122)	ns
AST (U / l)	20 (13–33)	23 (18–40)	ns
ALT (U / l)	18 (7–26)	22 (13–50)	ns
AST/ALT-ratio	1.4 (0.8–2.3)	1.1 (0.7–1.4)	ns
AP (U / l)	69 (41–94)	72 (44–94)	ns
GGT (U / l)	15 (8–123)	22 (12–53)	ns
Total cholesterol (mg / dl)	167 (148–207)	158 (124–261)	ns
Triglycerides	77 (46–101)	121 (60–175)	ns
Fat mass (BIA) (kg)	16.4 (7.6–31.8)	18.8 (9.5–42.3)	ns
Fat free mass (BIA) (kg)	58.4 (43.7–72.0)	58.5 (43.2–63.5)	ns
Body cell mass (BIA) (kg)	31.6 (22.5–39.0)	27.9 (20.4–33.0)	ns
Phase angle (BIA) (degrees)	6.7 (6.1–6.9)	5.6 (4.7–7.2)	ns
Glucose during clamp			
hyperglycemia	267 (212–367)	267 (196–449)	ns
euglycemia	87 (72–105)	93 (84–97)	ns
hypoglycemia	58 (47–70)	58 (51–66)	ns

Given are absolute numbers for gender as well as the number of smokers and median values and/or ranges for all other parameters. BMI = body mass index; HbA1c = glycated hemoglobin A1c; mmol = millimol; uACR = urinary albumin-creatinine-ratio; NSS = neuropathy symptom score; NDS = neuropathy deficit score; hsCRP = high sensitive c-reactive protein; eGFR = estimated glomerular filtration rate; CKD-EPI = Chronic Kidney Disease Epidemiology Collaboration; AST = aspartate aminotransferase; ALT = alanine aminotransferase; AP = alkaline phosphatase; GGT = gamma-glutamyl transferase; HDL = high-density lipoprotein; LDL = low-density-lipoprotein; m = male; f = female; kg = kilograms; m^2^ = square meters; mg = milligrams; g = grams; ml = milliliters; min = minute; mU = milliunits; l = liters; kPa = kilopascals; IQR = interquartile range; l = liters; dl = deciliters; U = units.

### Baseline testing

**Fig 1:**
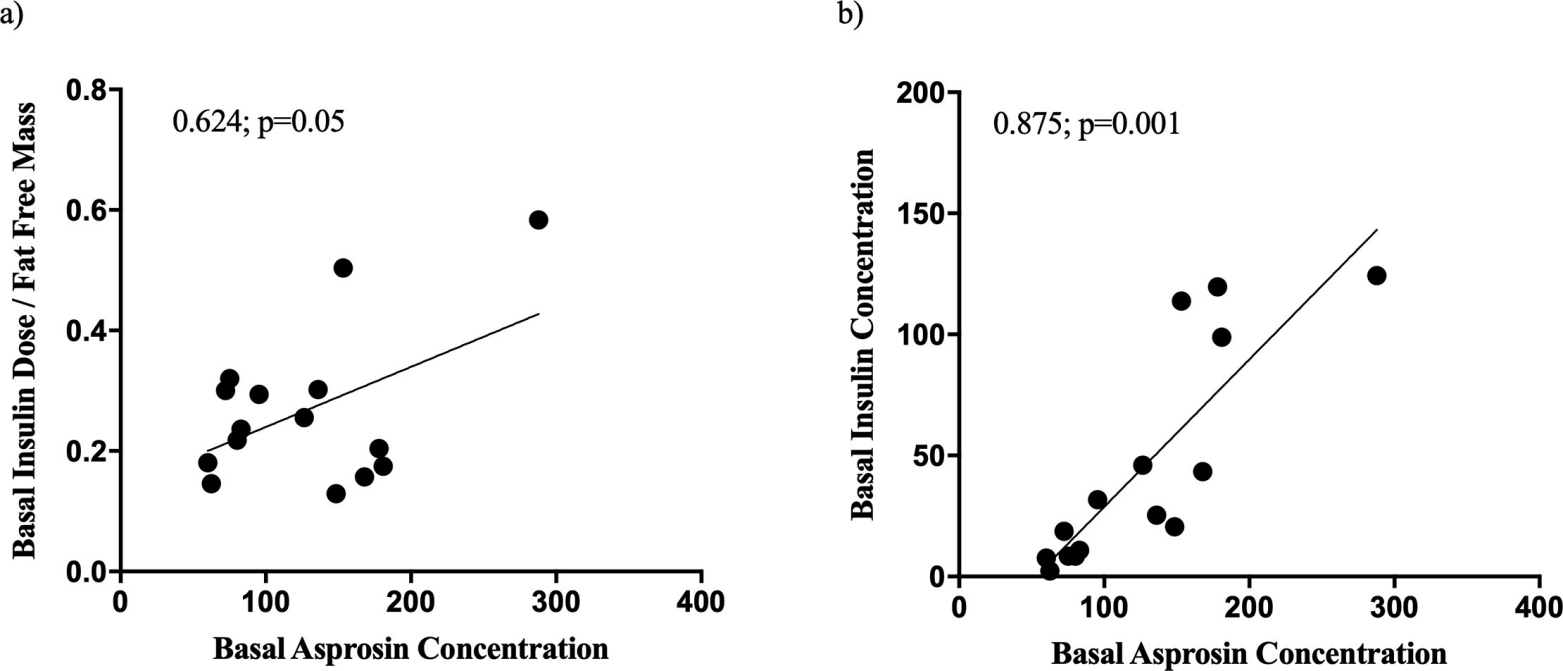
Baseline correlation analysis. Correlations between asprosin concentrations in plasma and surrogate parameters for insulin resistance at baseline before the clamp study. (a) Trend towards a correlation between asprosin levels and basal insulin dose normalized to fat-free mass. (b) Correlation between asprosin levels and serum insulin concentrations. All patients had no sufficient endogenous insulin production, therefore, serum insulin concentrations represented injected basal insulin concentrations. Shown are Spearman Correlation Coefficients and p-values. Basal insulin dose is given in units per day. Insulin concentrations are given in milliunits per liter.

Initial plasma asprosin levels in a fasting state prior to the test correlated positively with serum insulin concentrations (spearman correlation coefficient 0.875; p = 0.001) and a trend towards a positive correlation with patients' regular basal insulin doses normalized to fat-free mass could be found (spearman correlation coefficient 0.624; p = 0.054) (Fig 1).

### Intravenous glucose tolerance test and hypoglycemic hyperinsulinemic clamp test

**Fig 2:**
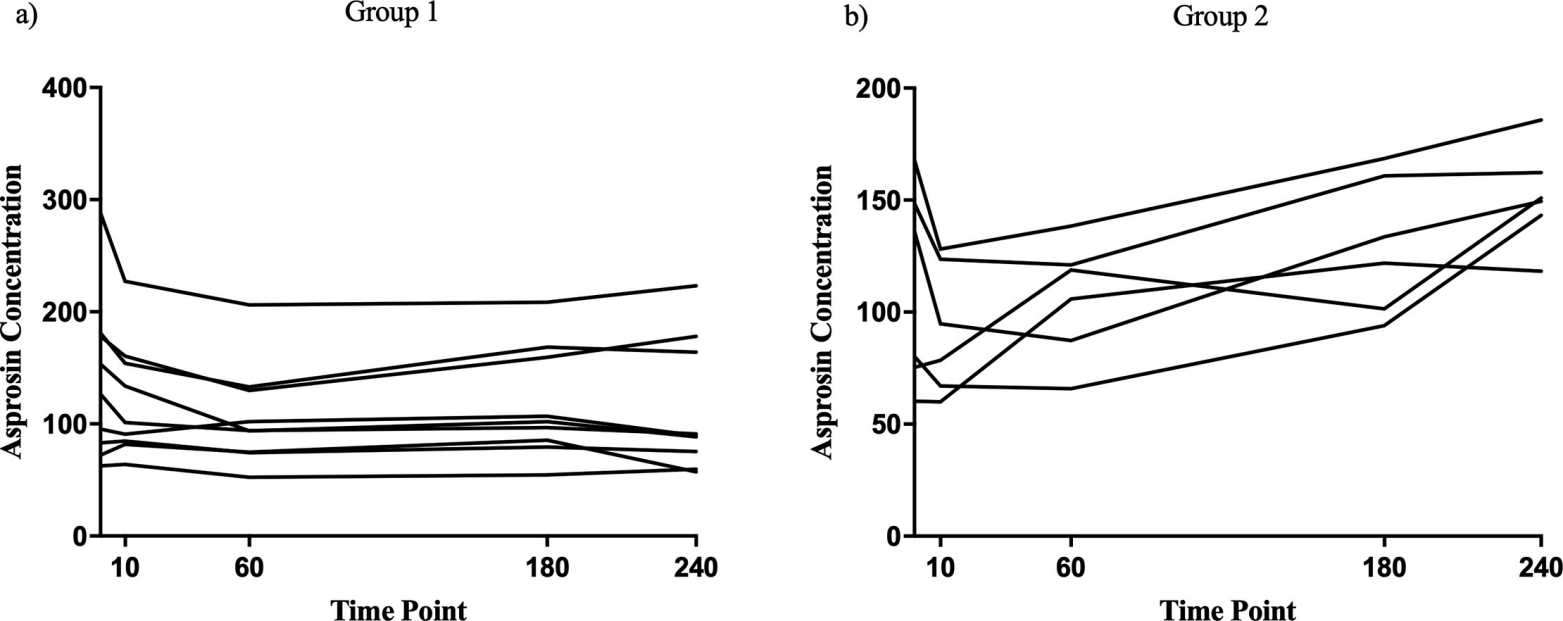
Individual asprosin concentrations. Individual asprosin concentrations during the ivGTT and clamp study. X-Axis shows the time points in minutes. 0 = baseline before the ivGTT was started, median blood glucose level was 159 mg/dl. 10 = ten minutes after intravenous glucose injection, median blood glucose level was 267 mg/dl. 60 = 60 minutes after intravenous glucose injection, median blood glucose level was 227 mg/dl. 180 = during the hyperinsulinemic clamp at a median glucose level of 89 mg/dl in a steady state. 240 = during the hyperinsulinemic clamp at a median glucose level of 58 mg/dl in a steady state. There was no statistical difference between the absolute values of asprosin levels at the different time points. However, the delta values between baseline asprosin levels and asprosin levels during hypoglycemia were significantly different between the groups (p<0.001). Asprosin concentrations are given in nanograms per milliliter. (a) Individual asprosin concentrations of patients without increase in asprosin concentrations during initial blood glucose lowering or hypoglycaemia (group 1, n = 9). (b) Individual asprosin concentrations of patients with increase in asprosin concentrations during initial blood glucose lowering or hypoglycaemia (group 2, n = 6). ivGTT = intravenous glucose tolerance test. mg/dl = milligrams per deciliter.

Six patients showed increasing levels of asprosin during the blood glucose lowering after ivGTT or the hypoglycemic phase of the clamp test, whereas nine patients did not show such an increase (group comparison of deltas of asprosin levels between baseline and hypoglycemia p<0.001, Fig 2). Asprosin levels did not differ at baseline and were not significantly different under hyper-, normo-, or hypoglycemia when comparing these two groups. There were no significant differences in asprosin plasma levels and no differences in changes of asprosin plasma concentrations during hypoglycemia between patients with and without hypoglycemia unawareness (initial fasted asprosin levels 95 vs 130 ng/ml, asprosin levels during hyperglycemia 91 vs 115 ng/ml, asprosin levels during hypoglycemia 118 vs 147 ng/ml, respectively). Since hypoglycaemia unawareness did not explain the heterogeneity of the asprosin release, alternative parameters were sought.

**Fig 3:**
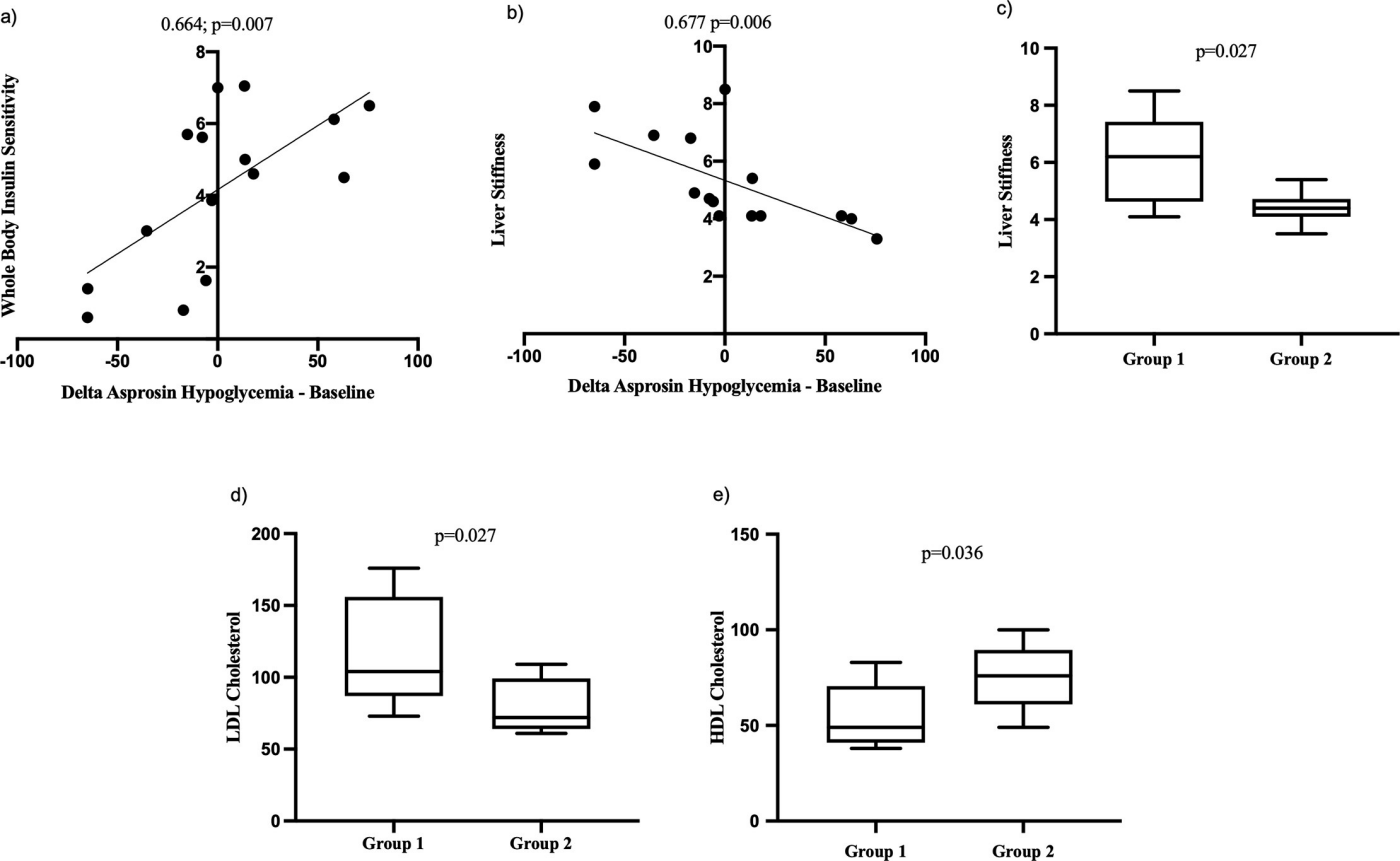
Clinical parameters associated with asprosin increase during hypoglycemia. Group 1 consists of patients without asprosin increase during initial blood glucose lowering or the hypoglycemic phase of the clamp study, group 2 consists of patients with such an increase. (a) shows the correlation between calculated whole-body insulin sensitivity and the calculated delta in asprosin plasma concentrations between the hypoglycemic phase and baseline values. (b) shows the correlation between liver stiffness measured by FibroScan^®^ and the calculated delta in asprosin plasma concentrations between the hypoglycemic phase and baseline values. (c) shows the difference in liver stiffness values measured by FibroScan^®^ between groups 1 and 2. (d) shows the difference in LDL cholesterol levels between groups 1 and 2. (e) shows the difference in HDL cholesterol levels between groups 1 and 2. Calculated whole-body insulin sensitivity is given in milligrams glucose / kilograms fat-free mass x minute. The delta in asprosin plasma concentrations between the hypoglycemic phase and baseline values is given in nanograms per milliliter. Liver stiffness is given in kilopascals. LDL and HDL cholesterol are given in milligrams per deciliters. LDL = low-density lipoprotein. HDL = high-density lipoprotein. With correlations, spearman correlation coefficient is shown.

Comparing patients with and without an increase in asprosin levels during initial blood glucose lowering or hypoglycemia, patients with an increase of asprosin showed higher calculated whole-body insulin sensitivity (p = 0.050), and there were significant positive correlations between calculated whole-body insulin sensitivity and the calculated delta values of plasma asprosin between the hypoglycemic phase and the baseline (spearman correlation coefficient 0.664; p = 0.007, Fig 3B) as well as asprosin increase (spearman correlation coefficient 0.535; p = 0.04). Moreover, patients without increase in asprosin levels had significantly higher liver stiffness values compared to patients with asprosin increase during the hypoglycemic phase (p = 0.026, Fig 3C), and higher liver stiffness was significantly associated with lower calculated delta values of plasma asprosin between the hypoglycemic phase and baseline (spearman correlation coefficient -0.677; p = 0.006, Fig 3B) as well as with impaired asprosin increase during hypoglycemia (spearman correlation coefficient -0.599; p = 0.018). Patients with higher values for liver stiffness in ultrasound elastography also showed steatosis in B-mode ultrasound (median 5.6 kPa in these patients). Patients without an asprosin increase showed significantly higher LDL levels (104 vs. 72 mg/dl, Fig 3D) and lower HDL levels (49 vs. 76 mg/dl, p = 0.036, Fig 3E) as compared to patients with an asprosin increase. Additionally, a blunted asprosin increase correlated significantly with lower HDL levels (spearman correlation coefficient 0.568; p = 0.027) and higher LDL levels (spearman correlation coefficient -0.583; p = 0.022), and the delta values of plasma asprosin between the hypoglycemic phase and the baseline correlated correlated significantly with both HDL (spearman correlation coefficient 0.533; p = 0.041) and LDL levels (spearman correlation coefficient -0.540; p = 0.038).

Changes in asprosin levels did not correlate with microvascular diabetic complications (retinopathy, nephropathy, peripheral, or autonomic neuropathy), hsCRP, HbA1c, age, gender, AST, ALT, AST/ALT-ratio, and diabetes duration. As expected, no patient showed a significant increase in C-peptide concentrations during the ivGTT.

## Discussion

The expected physiological asprosin response in hypoglycemia proved to be blunted in patients with diabetes mellitus type 1 who showed signs of insulin resistance. As marker for insulin resistance, calculated whole-body insulin sensitivity was used and was found to be lower in patients without an increase of asprosin during hypoglycemia. The method for calculation of insulin sensitivity has been described before in a high-ranking publication in patients with diabetes mellitus type 1 [16], although at a blood glucose level of 90 mg/dl. No studies on the validation of HOMA index in diabetes mellitus type 1 could be found. As this calculation was only relevant for group comparison, the euglycemic phase was too short to reach a meaningful steady-state, and there are no valid methods for evaluating insulin sensitivity during hypoglycaemia, this calculation of whole-body insulin sensitivity was chosen as the closest approximation to evaluate insulin sensitivity in this cohort.

Moreover, the missing asprosin increase in these patients was most likely associated with the beginnings of non-alcoholic fatty liver disease (NAFLD), since they showed slightly higher values for liver stiffness in ultrasound elastography combined with the beginning of steatosis in B-mode ultrasound. This statement is emphasized by the fact that all four patients with pathological values for liver stiffness [18] (three >6.7 kPa, one with 8.5 kPa) showed a blunted asprosin response during hypoglycemia. NAFLD is associated with insulin resistance and is common in patients with diabetes mellitus type 2 [19, 20]. For patients with diabetes mellitus type 1, no data on the association of insulin resistance and NAFLD could be found in the literature at the time of writing, although NAFLD has been observed in patients with diabetes type 1 before, with a prevalence of approximately 30% [19]. Additionally, imbalanced adipokine profiles have been associated with metabolic inflammation in NAFLD, potentially even causing hepatocellular carcinoma [21]. Since the data presented in this pilot study support an association between asprosin and NAFLD, insulin resistance and NAFLD seem to play a significant role in non-obese patients with diabetes mellitus type 1 and are likely underestimated in clinical routine.

Furthermore, patients with higher LDL and lower HDL levels did not show the expected increase in asprosin concentrations during hypoglycemia in the clamp test. Again, a significant positive association between dyslipidemia and NAFLD in adult males has been shown previously [22, 23]. In addition to that, studies have shown an association between plasma levels of asprosin and triglycerides (in patients with diabetes mellitus type 2 as well as persons without diabetes), as well as HDL levels (in persons without diabetes) [24]. Therefore, not only circulating plasma levels of asprosin, but also changes in asprosin plasma concentrations or asprosin release seem to be associated with these parameters.

Nevertheless, the data presented indicates that hypoglycaemia unawareness cannot be explained by a lack of asprosin release under hypoglycemic conditions as neither the changes in asprosin levels at different blood glucose concentrations, nor asprosin plasma levels during hypoglycaemia differed between patients with and without hypoglycaemia unawareness. In our study, patients with hypoglycemia unawareness had diabetes for at least 20 years. This is in accordance with previous literature, which showed that diabetes duration is a risk factor for the development of hypoglycemia unawareness [8]. All patients reached hypoglycemic target levels, and the rises in plasma concentrations of adrenalin or noradrenalin indicated significant stress during hypoglycemia at least in seven of the ten patients, showing the effectiveness of the clamp study to simulate hypoglycemic stress. Moreover, since some of the patients with very high increases in catecholamine plasma levels did not show rises in asprosin plasma levels, the asprosin release does not seem to be regulated by the same adreno-cortical systems.

The main limitation of this study is the small number of participants, since it was only designed as a small pilot project. However, changes in asprosin plasma levels under different fasting states are quite significant in previously published literature [1]. Consequently, similarly changes in the plasma levels of asprosin under different glucose concentrations would have been expected, especially in case of asprosin playing a significant role in regulatory mechanisms during hypoglycemia. It was therefore expected that the number of patients would have been sufficient for observing differences. The range of diabetes duration was quite high, although only one patient had been recently diagnosed (two years before the study), while all other patients had a history of more than 10 years of diabetes mellitus type 1. Therefore, patients in this study mostly had long-standing diabetes.

To date, this is the first study focusing on asprosin in patients with diabetes mellitus type 1. Interestingly, the relevance of asprosin seems to be similar in diabetes mellitus types 1 and 2, since in both, asprosin concentrations are associated with insulin resistance as well as dyslipidemia. Moreover, at least in diabetes type 1, alterations in liver structure, most likely early stages of NAFLD are associated with asprosin levels. This study shows that these clinical parameters are not only associated with elevated plasma levels of asprosin, but also to a blunted asprosin response in hypoglycemia, whereas no other clinical parameters associated with asprosin concentrations under different glucose concentrations could be found. These results would suggest that insulin resistance, dyslipidemia, and NAFLD seem to play a role even in non-obese patients with diabetes mellitus type 1 to an extent which is associated with changes in plasma levels and response of a glucogenic hormone. Since the interactions between adipokines, insulin resistance, and NAFLD might be relevant for future therapeutic interventions and also play a role in diabetes type 1, the association between asprosin and NAFLD including inflammatory processes should be examined in more detail in future studies.

## Supporting information

S1 Table Inclusion criteria. Shown are the inclusion criteria for participants without and with hypoglycemia unawareness. oGTT = oral glucose tolerance test.
(PDF)

S2 Table Exclusion criteria. Shown are the exclusion criteria for all participants, regardless of hypoglycemia unawareness. ADA = American Diabetes Association; NYHA = New York Heart Association; PBC = primary biliary cirrhosis; PSC = primary sclerosing cholangitis, HIV = human immunodeficiency virus; ICD = implantable cardioverter-defibrillator.
(PDF)

S3 Table Raw data table. Shown is the raw data acquired during the study and used for statistical analysis and interpretation of the results presented in this manuscript.
(PDF)
