# Supplementary material for: Asprosin response in hypoglycemia is not related to hypoglycemia unawareness but rather to insulin resistance in type 1 diabetes
Source: PLoS One. 2019 Sep 19;14(9):e0222771. doi: 10.1371/journal.pone.0222771 (PMC6752946; doi:10.1371/journal.pone.0222771)
Supplement: S1 Table — Shown are the inclusion criteria for participants without and with hypoglycemia unawareness. oGTT = oral glucose tolerance test. (PDF) [file pone.0222771.s001.pdf]

Supporting information

S1 Table. Inclusion criteria.

Inclusion criteria for participants without hypoglycemia unawareness

- Age between ≥ 18 and ≤ 75 years
- BMI between 20 und 35 kg/m^2
- Persons with manifest diabetes mellitus type 1 and diagnosis according to DDG guidelines 2018 oGTT, HbA1c ≥ 6.5% in the absence of adulteration of the HbA1c, over 200 mg / dl in the 2 hour value of the oGTT, fasting glucose > 126 mg/dl, spontaneous glucose > 200 mg/dl at least twice).

Inclusion criteria for participants with hypoglycemia unawareness

- Persons who are not aware of hypoglycemia symptoms at glucose levels below 50 mg / dl
- Age between ≥ 18 and ≤ 75 years
- BMI between 20 und 35 kg/m^2
- Persons with manifest diabetes mellitus type 1 and diagnosis according to DDG guidelines 2018 oGTT, HbA1c ≥ 6.5% in the absence of adulteration of the HbA1c, over 200 mg / dl in the 2 hour value of the oGTT, fasting glucose > 126 mg/dl, spontaneous glucose > 200 mg/dl at least twice.
