# Supplementary material for: Asprosin response in hypoglycemia is not related to hypoglycemia unawareness but rather to insulin resistance in type 1 diabetes
Source: PLoS One. 2019 Sep 19;14(9):e0222771. doi: 10.1371/journal.pone.0222771 (PMC6752946; doi:10.1371/journal.pone.0222771)
Supplement: S2 Table — Shown are the exclusion criteria for all participants, regardless of hypoglycemia unawareness. ADA = American Diabetes Association; NYHA = New York Heart Association; PBC = primary biliary cirrhosis; PSC = primary sclerosing cholangitis, HIV = human immunodeficiency virus; ICD = implantable cardioverter-defibrillator. (PDF) [file pone.0222771.s002.pdf]

S2 Table. Exclusion criteria.

General exclusion criteria

- Secondary types of diabetes (ADA-criteria type 3 B-H)
- Current pregnancy
- Acute infections / fever
- Immune-suppressant therapy
- Severe psychiatric diseases requiring treatment (for example personality disorders, schizophrenia, depression)
- Known alcohol or drug dependency
- Severe heart-, kidney-, or liver-insufficiency:
    - NYHA stadium IV
    - Non-diabetic liver disease (for example PBC, PSC, Wilson's disease, hemochromatosis, autoimmune hepatitis)
    - severe peripheral artery disease (stadium IV)
    - non-diabetic glomerulopathy
- Cancer or other malignant diseases within the last 5 years
- Infectious diseases like hepatitis B, C, E, or HIV
- Other severe autoimmune diseases
- Current participation in an interventional study
- Anemia or disorders of bone marrow
- Exclusion criteria for clamp study
- Past history of deep vein thrombosis or pulmonary embolism
- Routine lab results ≤ 80% below lower reference value: Ferritin, iron, leucocytes, haemoglobin, hematocrit, RBC, platelets, blood alcohol levels.
- Exclusion criteria for bioimpedance measurement
- Pacemaker / ICD
- Exclusion criteria for lung function testing
- Ignoring or non-understanding of the instructions
