# Supplementary material for: Asprosin response in hypoglycemia is not related to hypoglycemia unawareness but rather to insulin resistance in type 1 diabetes
Source: PLoS One. 2019 Sep 19;14(9):e0222771. doi: 10.1371/journal.pone.0222771 (PMC6752946; doi:10.1371/journal.pone.0222771)
Supplement: S3 Table — Shown is the raw data acquired during the study and used for statistical analysis and interpretation of the results presented in this manuscript. (PDF) [file pone.0222771.s003.pdf]

S3 Table. Complete raw data.

Number	Age	Gender	G20 infusion rate	WB insulin sensitivity
1	60,00	0	80,00	1,72
2	51,00	1	140,00	2,11
3	29,00	0	10,00	,18
4	50,00	1	120,00	1,81
5	30,00	0	80,00	1,83
6	37,00	1	60,00	1,02
7	54,00	1	100,00	1,69
8	47,00	1	30,00	0,49
9	56,00	0	50,00	1,16
10	52,00	0	65,00	1,29
11	75,00	0	60,00	0,94
13	30,00	1	75,00	1,60
14	58,00	1	10,00	0,22
15	72,00	0	150,00	2,56
16	66,00	0	130,00	1,81

Number	Glucose baseline	Glucose 4 min	Glucose 10 min	Glucose 60 min	Glucose target 90	Glucose target 60
1	153,00	262,00	232,00	185,00	94,00	55,00
2	174,00	288,00	262,00	219,00	93,00	55,00
3	196,00	356,00	336,00	255,00	89,00	59,00
4	97,00	205,00	212,00	193,00	89,00	47,00
5	141,00	384,00	367,00	323,00	84,00	60,00
6	128,00	249,00	259,00	193,00	97,00	56,00
7	159,00	300,00	267,00	235,00	93,00	56,00
8	231,00	348,00	342,00	282,00	84,00	60,00
9	254,00	389,00	402,00	358,00	96,00	66,00
10	202,00	330,00	319,00	284,00	84,00	67,00
11	132,00	277,00	256,00	214,00	94,00	51,00
12	178,00	276,00	272,00	227,00	105,00	70,00
13	303,00	482,00	449,00	404,00	87,00	65,00
14	99,00	228,00	196,00	167,00	85,00	58,00
15	99,00	281,00	231,00	195,00	72,00	54,00

Number	Basal insulin dose	Ins baseline	Insulin 4'	Insulin 10'	Insulin 60'	Insulin at glucose 90	Insulin at glucose 60
1	14,00	18,70	13,30	14,50	13,30	70,30	70,90
2	20,00	25,40	19,40	24,50	21,30	126,90	127,20
3	27,80	113,80	108,90	101,10	88,30	149,50	138,60
4	12,00	7,70	6,90	7,40	7,30	93,60	81,50
5	14,00	8,40	8,00	8,20	8,10	118,00	114,30
6	15,00	46,00	45,90	47,20	51,90	136,20	120,90
7	14,00	10,90	10,70	9,30	8,10	48,00	56,70
8	18,00	31,80	28,50	27,70	24,40	88,10	83,50
9	6,30	2,30	1,50	1,50	,80	37,00	32,10
10	11,00	8,40	8,50	8,10	6,70	55,10	56,70
11	18,00	124,30	122,40	122,00	124,80	211,90	183,40
12	5,50	43,40	34,10	34,50	32,90	117,50	134,00
13	7,90	98,90	88,70	83,30	93,80	201,20	221,20
14	6,10	119,60	92,90	92,10	96,70	191,60	199,90
15	9,30	20,60	19,90	18,90	19,10	169,30	155

Number	c-pept baseline	c-pept 4'	c-pept 10'	c-pept 60'	c-pept at gluc 90	c-pept at gluc 60
1	,10	,10	,10	,10	,10	,10
2	,10	,10	,10	,10	,10	,10
3	,10	,10	,10	,10	,10	,10
4	,10	,10	,10	,10	,10	,10
5	,10	,10	,10	,10	,10	,10
6	,50	,50	,60	,80	,50	,30
7	,10	,10	,10	,10	,10	,10
8	,10	,10	,10	,10	,10	,10
9	,10	,10	,10	,10	,10	,10
10	,30	,40	,40	,40	,10	,10
11	,10	,10	,10	,10	,10	,10
12	,10	,10	,10	,10	,10	,10
13	,10	,10	,10	,10	,10	,10
14	,10	,10	,10	,10	,10	,10
15	,10	,10	,10	,10	,10	,10

Number	Asprosin baseline	Asprosin 10'	Asprosin 60'	Asprosin at gluc 90	Asprosin at gluc 60
1	72,26	81,90	74,72	85,44	57,13
2	136,10	94,75	87,33	133,60	149,49
3	153,32	133,76	93,75	102,14	88,37
4	60,18	59,99	105,85	121,94	118,32
5	75,27	78,48	118,76	101,42	151,02
6	126,51	101,25	93,93	96,86	91,06
7	82,99	84,82	74,21	79,31	75,32
8	95,43	90,79	102,19	106,85	89,59
9	62,53	63,79	52,44	54,60	59,65
10	80,19	67,02	65,90	93,98	143,27
11	287,98	227,31	206,20	208,52	223,09
12	167,93	128,12	138,38	168,64	185,80
13	180,95	154,15	133,12	168,38	163,87
14	178,02	160,52	129,70	159,40	178,10
15	148,54	123,61	121,14	160,78	162,25

Number	Cortisol baseline	Cortisol 60'	Cortisol at gluc 60	ACTH baseline	ACTH 60'	ACTH at gluc 60
1	176,50	98,80	37,80	16,40	10,50	9,10
2	163,20	105,20	61,20	23,60	17,90	16,50
3	168,30	171,60	133,10	21,20	28,90	24,40
4	87,40	59,20	67,70	11,20	8,90	9,80
5	192,60	111,50	83,90	20,00	12,90	15,30
6	255,00	113,00	46,80	18,30	5,00	6,60
7	101,00	69,40	93,40	8,10	5,40	10,30
8	125,10	88,00	76,00	14,70	13,60	19,30
9	128,70	65,00	75,80	17,40	12,20	20,70
10	179,30	113,50	75,00	12,80	9,00	8,50
11	107,30	96,20	96,70	32,20	26,20	24,20
12	199,20	112,90	42,20	20,70	13,10	9,30
13	148,70	123,20	84,10	14,50	14,90	13,90
14	185,40	143,80	87,80	32,10	17,20	17,50
15	168,90	104,80	80,90	22,30	15,30	13,70

Number	Glucagon baseline	Glucagon 60'	Glucagon at gluc 60	NA baseline	NA 60'	NA at gluc 60
1	124,00	119,00	141,00	1255,00	1857,00	1956,00
2	97,00	89,00	87,00	2067,00	1895,00	2037,00
3	119,00	138,00	118,00	720,00	670,00	918,00
4			96,00	1132,00	1566,00	2091,00
5	126,00	142,00	123,00	2090,00	1589,00	2283,00
6	97,00	91,00	72,00	2120,00	2314,00	3844,00
7	78,00	86,00		996,00	927,00	905,00
8	125,00	108,00	114,00	896,00	1105,00	1903,00
9	132,00	109,00	91,00	1991,00	2833,00	2677,00
10	125,00	128,00	117,00	1400,00	2174,00	2299,00
11				3460,00	2344,00	3406,00
12				2169,00	1621,00	1330,00
13				1611,00	1314,00	2358,00
14				2947,00	1805,00	3265,00
15				1875,00	1624,00	2511,00

Number	Adr baseline	Adr 60'	Adr at gluc 60	dopa baseline	dopa 60'	dopa at gluc 60
1	695,00	82,00	82,00	837,00	1509,00	302,00
2	567,00	404,00	430,00	1206,00	1541,00	1327,00
3	382,00	82,00	375,00	2739,00	2505,00	1141,00
4	526,00	89,00	641,00	553,00	365,00	1220,00
5	516,00	231,00	82,00	1971,00	1097,00	361,00
6	600,00	621,00	411,00	494,00	677,00	770,00
7	278,00	82,00	333,00	474,00	1100,00	810,00
8	82,00	82,00	1235,00	3864,00	890,00	2973,00
9	518,00	444,00	539,00	1195,00	1045,00	975,00
10	82,00	198,00	224,00	1222,00	389,00	321,00
11	272,90	272,90	385,00	391,90	391,90	391,90
12	456,00	301,00	272,90	976,00	497,00	391,90
13	272,90	272,90	605,00	533,00	420,00	391,90
14	272,90	272,90	272,90	391,90	391,90	552,00
15	285,00	272,90	407,00	1110,00	808,00	391,90

Number	TSH	Fibroscan kap	Fibroscan IQR	lungdisease	PAD	ABI right	ABI left	Thyroiddisease	Retinopathy Photo	Smoking
1	,37	4,80	1,00	,00	,00	,94	1,11	1,00	1,00	,00
2	1,57	4,30	,60	1,00	,00	1,11	1,09	,00	,00	,00
3	1,13	5,60	1,00	,00	,00	1,10	1,02	,00	,00	,00
4	1,16	4,40	,80	,00	,00	1,22	1,10	,00	,00	,00
5	,18	3,50	1,10	,00	,00	,99	1,02	1,00	,00	,00
6	1,05	6,90	,70	,00	,00	1,05	1,07	,00	,00	1,00
7	1,87	4,70	,20	,00	,00	1,10	1,14	,00	1,00	,00
8	5,11	4,60	,40	,00	,00	1,08	1,06	1,00	1,00	,00
9	2,23	4,10	1,10	,00	,00	1,09	1,01	,00	,00	,00
10	1,69	4,50	1,50	,00	,00	1,10	1,17	,00	,00	,00
11	2,47	7,60	,80	,00	,00	1,18	1,15	,00	,00	,00
12	1,42	4,40	,90	,00	,00	1,15	1,18	1,00	,00	,00
13	1,41	6,80	,60	,00	,00	,95	,97	,00	,00	1,00
14	2,35	8,50	9,30	,00	,00	1,13	1,07	,00	1,00	,00
15	,31	5,40	,60	,00	,00	1,14	1,16	1,00	,00	,00

Number	height	weight	BMI	Hb	MCV	MCH	MCHC	Crea	GFRCKDEPI	LDL	HDL	totChol	TAG	Lp(a)
1	176	65	21	11,90	91	31	35	,67	95,80	104	83	213	128	10,00
2	179	85	26,5	13,60	94	32	35	,70	109,30	65	86	169	89,00	10,00
3	171	86,6	29,6	14,70	88	30	34	,62	122,20	176	50	261	174,00	87,14
4	182	74	22,3	13,40	92	31	34	,94	94,20	61	100	170	46	15,97
5	159	72	28,5	11,80	87	28	32	,54	127,00	71	65	148	60,00	73,05
6	178	70	22,1	14,50	88	32	36	,86	110,80	97	42	153	72,00	10,00
7	170	75	26	14,20	94	33	35	,79	101,80	85	49	158	121,00	29,23
8	174	84,6	27,9	14,60	92	32	35	,87	102,80	167	40	242	175,00	10,00
9	164	62	23,1	12,50	88	30	34	,62	101,10	145	82	239	60,00	10,00
10	173	63	21	12,50	91	30	33	1,06	60,10	109	83	207	73	10,00
11	184	86	25,4	12,30	89	31	35	1,56	42,80	110	38	151	61	85,04
12	174	61	20,1	13,10	89	31	34	,64	120,10	73	69	148	80	39,71
13	150	87,5	38,9	11,60	88	32	37	,67	97,20	73	59	158	130	
14	177	68	21,7	12,10	87	29	33	,78	90,20	89	45	124	74	10,66
15	182	103,8	31,3	14,90	84	29	35	,81	92,60	96	49	165	101	10,00

Number	hsCRP	ntproBNP	PTH	Iron	Tranfsat	Ferritin	GOT	GPT	AP	GGT	HbA1c	uACR	RRsys	RRdia
1	,16	230	2,80	12,60	25	44	25	23	81	15	7,9	,70	110	70
2	2,89	40	6,00	9,10	15	166	33	25	94	42	9,0	,35	121	78
3	7,93	53	1,60	14,00	25	56	23	22	76	23	8,0	2,50	116	68
4	,41	40	2,30	12,30	24	112	21	23	66	17	5,9	3,00	116	73
5	12,40	23	3,00	10,60	22	105	13	9	71	10	7,5	,37	130	86
6	,41	42	2,10	27,40	52	48	19	16	44	12	6,5	3,00	118	78
7	1,02	23	8,20	11,90	19	102	23	21	72	20	7,6	1,36	160	94
8	4,14	59	4,40	8,80	18	152	40	50	94	53	8,5	3,00	129	80
9	,68	140	3,40	11,20	26	110	27	20	73	22	7,5	3,00	136	83
10	,60	108	8,80	13,60	31	55	19	12	48	8	6,5	1,11	126	84
11	6,03	319	8,50	14,70	28	175	18	13	58	12	7,5	4,03	120	66
12	,15	31	3,70	19,80	29	19	16	7	41	12	6,3	8,38	110	74
13	5,20	42	7,80	16,90	27	322	23	33	64	41	8,4	61,25	158	87
14	1,86	244	6,20	14,20	27	237	33	24	65	45	7,5	3,19	123	69
15	3,66	71	5,50	16,30	38	396	21	26	85	123	7,5	3,92	139	79

Number	hypo unawareness	diabetes duration	NSS	NDS	auton neurop.	statin	ASS	IMT right	IMT left
1	0	46,00	1	6	1,	1	1	,50	,50
2	1	26,00	0	2	0	1	0	,50	,40
3	0	22,00	0	0	0	0	0	,40	,40
4	1	24,00	0	0	0	0	0	,47	,59
5	0	19,00	0	0	0	0	0	,50	,50
6	0	2,00	0	0	0	0	0	,50	,50
7	1	30,00	0	2	0	1	0	,90	,50
8	1	20,00	4	0	0	0	0	,50	,60
9	1	31,00	0	2	0	0	0	,60	,60
10	0	36,00	0	0	0	0	0	,50	,60
11	1	51,00	5	4	0	1	1	,74	,62
12	0	12,00	0	0	0	0	0	,40	,40
13	0	22,00	0	2	0	0	0	,55	,65
14	0	34,00	0	6	0	1	0	1,17	1,05
15	1	20,00	6	10	0	0	0	,67	,96

Number	phase angle	BCM	FFM	Cells	Water balance	Fat mass	Body water	US steatosis
1	5,50	22,60	46,60	48,50	30,30	18,40	30,90	0
2	6,90	36,50	66,20	55,10	-20,70	18,80	48,90	0
3	6,80	30,20	55,20	54,70	-28,50	31,40	38,60	1
4	6,50	35,40	66,40	53,30	,00	7,60	48,50	1
5	6,10	22,50	43,70	51,60	-24,30	28,30	31,90	0
6	7,20	33,00	58,70	56,20	-49,50	11,30	41,50	1
7	6,90	32,60	59,30	54,90	-32,90	15,70	43,20	0
8	6,10	31,60	61,20	51,60	22,90	23,40	46,80	0
9	5,20	20,40	43,20	47,20	39,80	18,80	31,90	0
10	6,90	27,80	50,50	55,00	-51,20	12,50	34,60	0
11	4,70	27,90	63,50	44,00	171,30	22,50	47,10	0
12	6,70	25,50	47,00	54,20	-61,80	14,00	30,00	0
13	5,60	22,10	45,20	48,90	54,80	42,30	36,30	1
14	4,90	26,60	58,50	45,50	120,10	9,50	44,60	1
15	6,70	39,00	72,00	54,20	1,30	31,80	51,70	1
